# 

**Published:** 1878-09

**Authors:** 


### THE BUFFALO MEDICAL AND SURGICAL JOURNAL.

VOL. XVIII. SEPTEMBER, 1878. NO. 2.

